# Intraoperative hypotension burden and early postoperative recovery after uniportal video-assisted thoracoscopic lung resection: a retrospective cohort study

**DOI:** 10.3389/fmed.2026.1923645

**Published:** 2026-09-11

**Authors:** Yuyao Zhu, Lingyi Wei, Jianwen Zhou, Jingxiang Wu

**Affiliations:** 1 Department of Anesthesiology, Shanghai Chest Hospital, Shanghai Jiao Tong University School of Medicine, Shanghai, China; 2 Department of Anesthesiology, Shanghai Tongji Hospital, Tongji University School of Medicine, Shanghai, China

**Keywords:** intraoperative hypotension, mean arterial pressure, opioid consumption, postoperative nausea and vomiting, postoperative pain, video-assisted thoracoscopic surgery

## Abstract

**Objective:**

Intraoperative hypotension is common during general anesthesia and has been associated with postoperative organ injury. Its relationship with early postoperative analgesic requirement after uniportal video-assisted thoracoscopic surgery (VATS) remains unclear. This study aimed to evaluate whether cumulative intraoperative hypotension, defined primarily as duration of mean arterial pressure (MAP) <65 mmHg, is associated with postoperative analgesic consumption and early recovery outcomes after elective uniportal VATS lung resection.

**Methods:**

This single-center retrospective observational cohort study included 420 adult patients who had undergone elective uniportal VATS lung resection with standardized total intravenous anesthesia, thoracic paravertebral block, and postoperative intravenous patient-controlled analgesia (PCA). The primary exposure was cumulative duration of MAP < 65 mmHg, modeled per 10-min increase. The primary outcome was cumulative opioid consumption within 24 h postoperatively, standardized to intravenous morphine milligram equivalents. Secondary outcomes included resting pain, successful PCA dose deliveries, postoperative nausea and vomiting (PONV), and rescue analgesia. Multivariable regression models were used to evaluate adjusted associations.

**Results:**

Of 420 patients, 134 (31.9%) experienced at least one episode of MAP <65 mmHg. After full adjustment, each 10-min increase in MAP <65 mmHg duration was associated with higher 24-h opioid consumption [*β* = 1.513 mg; 95% confidence interval (CI), 0.877–2.149; *P* < 0.001], more successful PCA dose deliveries (*β* = 0.230; 95% CI, 0.124–0.336; *P* < 0.001), increased odds of PONV (OR, 1.63; 95% CI, 1.33–2.00; *P* < 0.001), and increased odds of rescue analgesic use (OR, 1.31; 95% CI, 1.10–1.58; *P* = 0.003). No significant association was observed with 24-h resting pain score.

**Conclusion:**

Greater cumulative duration of intraoperative MAP <65 mmHg was associated with higher early postoperative opioid requirement, greater analgesic demand, and increased PONV after uniportal VATS lung resection. These findings identify prolonged intraoperative hypotension as a potential marker of less favorable early recovery. Prospective studies are needed to determine whether the associations are causal and whether blood pressure-targeted interventions improve recovery.

## Graphical Abstract

**Figure:**
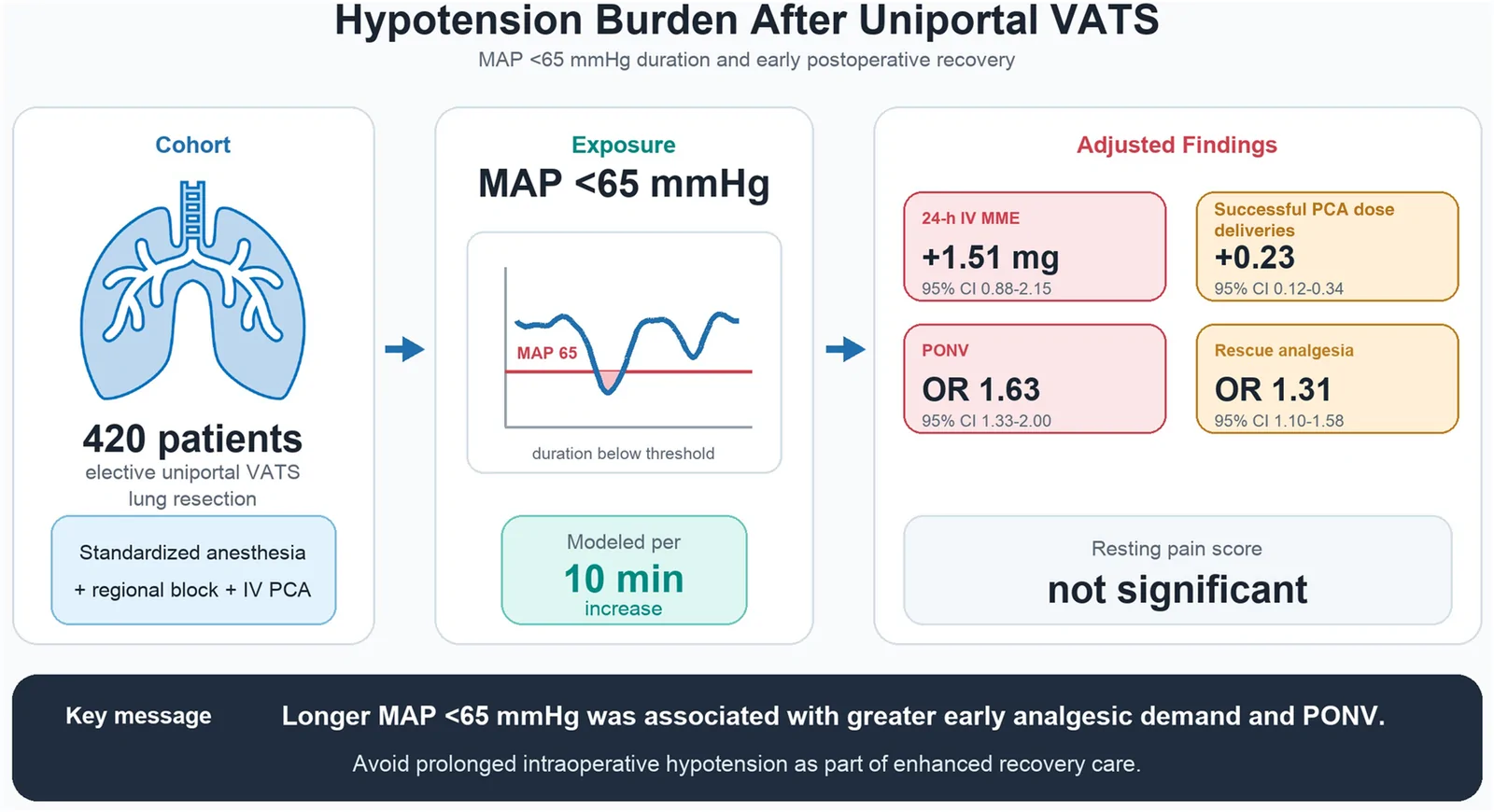

## Introduction

Lung resection performed through video-assisted thoracoscopic surgery (VATS) has become a central component of contemporary thoracic surgical practice and enhanced recovery pathways. Compared with thoracotomy, VATS is associated with less tissue trauma, improved early recovery, and lower postoperative pain in randomized and observational evidence. Nevertheless, clinically meaningful acute pain remains common after lung resection, even when minimally invasive and uniportal approaches are used (1–3). Thoracic surgical pain is complex because intercostal nerve irritation, pleural and chest tube stimulation, diaphragmatic irritation, and respiratory movement may all contribute to dynamic pain, impaired coughing, delayed mobilization, and greater opioid exposure (4, 5). In addition, acute postoperative pain after thoracic surgery is not only an early recovery endpoint but also a risk factor for persistent postsurgical pain, a complication reported after both thoracotomy and VATS (6, 7).

Current perioperative analgesic strategies for VATS therefore emphasize procedure-specific multimodal analgesia, opioid-sparing treatment, and regional techniques such as thoracic paravertebral block, erector spinae plane block, serratus anterior plane block, and intercostal nerve block (8–12). In spite of these advances, considerable interindividual variation persists in pain intensity, patient-controlled analgesia (PCA) use, and rescue analgesic requirement. Established determinants include surgical extent, baseline pain vulnerability, perioperative opioid exposure, and adequacy of regional analgesia. However, the contribution of intraoperative hemodynamic instability has received far less attention in thoracic analgesia research.

Intraoperative hypotension is common during general anesthesia, though its reported incidence varies substantially because definitions differ across studies (13, 14). Large perioperative cohorts and systematic reviews have linked even relatively brief or modest reductions in mean arterial pressure (MAP)—particularly below approximately 65 mmHg—with postoperative organ injury and adverse outcomes after non-cardiac surgery. Consensus statements increasingly emphasize both absolute pressure thresholds and cumulative hypotension burden, rather than isolated minimum blood pressure values, when evaluating perioperative risk. However, most existing work has focused on renal, myocardial, neurologic, or mortality outcomes; whether hypotension burden is associated with early postoperative pain, opioid consumption, PCA behavior, or postoperative nausea and vomiting (PONV) is less well characterized.

Several biologically plausible pathways have been proposed on the basis of related perioperative and pain literature rather than observations made directly in this cohort. Cumulative MAP reductions below approximately 65 mmHg have been associated with postoperative organ injury and may serve as a marker of perioperative physiologic stress (15–20). Inflammatory amplification, peripheral nociceptor sensitization, and central sensitization are established concepts in postsurgical pain biology (21–23), while remifentanil exposure may influence postoperative analgesic requirement through acute opioid tolerance or opioid-induced hyperalgesia (24–27). These mechanisms were not measured in the present study and are therefore considered hypotheses rather than demonstrated explanations.

This study was designed to evaluate whether cumulative intraoperative hypotension, defined primarily as duration of MAP <65 mmHg, is associated with postoperative analgesic consumption and early recovery outcomes after elective uniportal VATS lung resection under standardized anesthesia, thoracic paravertebral block, and PCA pathway. We hypothesized that greater hypotension burden would be associated with higher 24-h opioid consumption, more successful PCA dose deliveries, and higher PONV incidence.

## Materials and methods

### Study design and population

This was a single-center retrospective observational cohort study. Consecutive adult patients who underwent elective uniportal VATS wedge resection, segmentectomy, or lobectomy with postoperative intravenous patient-controlled analgesia between 1 January and 31 December 2025 were assessed for eligibility. Cases were identified from the institutional anesthesia information management system. Inclusion criteria required elective uniportal VATS lung resection, an available invasive arterial MAP time series from anesthesia start to end, use of the institutional postoperative PCA pathway, and ascertainment of 24-h postoperative outcomes. The analysis was restricted to the uniportal surgical approach, but the extent of lung resection remained heterogeneous and was included in the adjusted models. Postoperative follow-up for the study outcomes covered the first 24 h after surgery. Data were retrospectively extracted and reviewed between January and April 2026.

**Figure 1:**
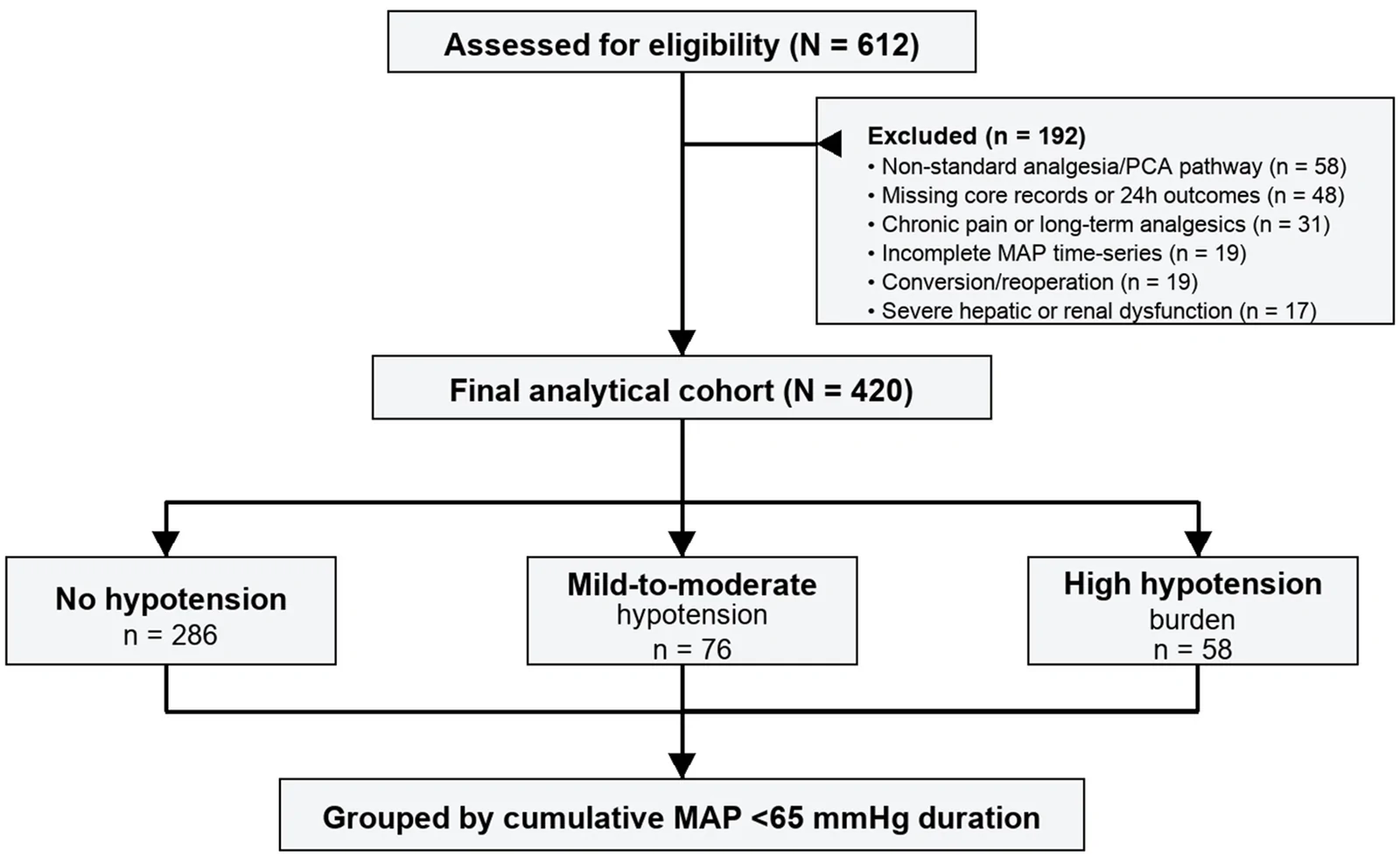
Study flowchart of patient selection and hypotension burden classification. Of 612 patients assessed, 192 were excluded and 420 were included (group sizes: *n* = 286, 76, and 58).

Exclusion criteria were missing core records or 24-h outcomes, incomplete intraoperative MAP time series, a non-standard postoperative analgesia pathway, conversion to thoracotomy, perioperative reoperation, severe hepatic or renal dysfunction, chronic pain conditions, or chronic opioid or neuropathic analgesic use. Of 612 patients assessed for eligibility, 192 were excluded: 58 for a non-standard analgesia/PCA pathway, 48 for missing core records or 24-h outcomes, 31 for chronic pain or long-term analgesic use, 19 for incomplete intraoperative MAP time series, 19 for conversion to thoracotomy or reoperation, and 17 for severe hepatic or renal dysfunction. The final analytical cohort comprised 420 patients (Figure 1).

### Standardized anesthesia, regional analgesia, and PONV prophylaxis

All patients were managed under the institutional perioperative pathway. Anesthesia was induced with propofol, sufentanil, and rocuronium, with doses individualized by the attending anesthesiologist. Anesthesia was maintained using conventional continuous intravenous infusions of propofol and remifentanil rather than target-controlled infusion. Sufentanil and additional rocuronium were administered according to clinical need. Routine bispectral index monitoring and train-of-four neuromuscular monitoring were not used. All patients underwent one-lung ventilation with a double-lumen endobronchial tube, with ventilatory settings individualized according to patient and intraoperative conditions.

All patients received an ultrasound-guided multilevel thoracic paravertebral block using 20 mL of 0.5% ropivacaine, performed by experienced anesthesiologists. Technical success was confirmed and documented by ultrasound visualization of correct needle-tip placement and local anesthetic spread within the thoracic paravertebral space in all included patients. A standardized postoperative sensory-level assessment was not available retrospectively. All patients received ondansetron 4 mg intravenously near the end of surgery for PONV prophylaxis.

Postoperatively, all patients received an electronic intravenous hydromorphone PCA pump. Hydromorphone was diluted with 0.9% saline to a total volume of 100 mL, and the total hydromorphone dose was individualized by the attending anesthesiologist according to the patient's clinical condition. Standard pump settings comprised a continuous background infusion of 2 mL/h, a patient-controlled bolus of 2 mL, and a 15-min lockout interval. Successful PCA dose deliveries were defined as doses actually delivered by the pump rather than all button presses. PCA was continued according to individual analgesic requirements for up to 48 h. When a patient reported inadequate analgesia despite PCA, the ward nurse performed a clinical assessment and administered intravenous tramadol 100 mg as rescue analgesia; no uniform numerical rating scale (NRS) threshold was imposed retrospectively.

### Surgical technique and chest drain management

The location and length of the uniportal incision and the extent of lung resection were selected by the operating surgeon according to patient’s anatomy, lesion characteristics, and the planned procedure. One 24-Fr chest drain was placed at the completion of surgery. Chest drain removal was determined by the surgical team according to air leakage, lung reexpansion, drainage characteristics and volume, and the patient's overall clinical status. No uniform numerical removal threshold was applied retrospectively. Exact chest drain duration was not available in the analytical dataset.

### Blood pressure time series processing and hypotension metrics

Intraoperative blood pressure time series data were extracted from the anesthesia information management system. All MAP measurements were obtained from invasive arterial pressure monitoring and stored at routinely charted, approximately 5-min intervals. Duplicate timestamps were deduplicated, and non-numeric, invalid, or system-flagged artifact records were excluded. Missing MAP intervals were not imputed. Cumulative duration of MAP below each specified threshold was calculated using the actual elapsed time between adjacent timestamped MAP records. A hypotension episode was defined as one or more consecutive valid MAP observations below the threshold; an intervening observation at or above the threshold or a missing interval ended the episode. The primary exposure was cumulative duration of MAP <65 mmHg, expressed in minutes and modeled per 10-min increase.

Secondary hypotension metrics included cumulative duration of MAP <60 mmHg, hypotension burden below 65 mmHg calculated as the pressure–time area below the 65-mmHg threshold (mmHg·min), time-weighted average hypotension below 65 mmHg, minimum MAP, number of hypotension episodes, and longest continuous duration of MAP <65 mmHg. For descriptive analyses, no hypotension was defined as a cumulative MAP <65 mmHg duration of 0 min, mild-to-moderate hypotension as a duration >0 and ≤15 min, and high hypotension burden as a duration >15 min. The 15-min boundary corresponded to the median positive hypotension duration among exposed patients.

### Outcomes and covariates

The primary outcome was cumulative opioid consumption within 24 h postoperatively, converted to intravenous morphine milligram equivalents (MME). All postoperative opioid exposure was standardized to intravenous morphine equivalents using parenteral equianalgesic ratios, with 10-mg intravenous morphine considered equivalent to 1.5-mg intravenous hydromorphone or 100-mg intravenous tramadol (28). Accordingly, 24-h intravenous MME was calculated as intravenous hydromorphone dose (mg) × 6.67 plus intravenous tramadol dose (mg) × 0.10. The hydromorphone dose was calculated from each patient's documented PCA concentration and actual pump-delivered volume. This outcome incorporated hydromorphone actually delivered through both the continuous background infusion and patient-controlled boluses, together with intravenous tramadol administered as rescue analgesia. Secondary outcomes included 24-h resting pain score, number of successful PCA dose deliveries within 24 h, PONV within 24 h, and rescue analgesic use. Resting pain was assessed once at the 24-h postoperative time point using an 11-point NRS, ranging from 0 (no pain) to 10 (worst imaginable pain). Dynamic pain during coughing or movement was not collected because of the retrospective study design. PONV was ascertained from Post-Anesthesia Care Unit (PACU) and ward nursing records and was defined as any documented episode of nausea, retching, or vomiting during the first 24 postoperative hours. Routine prophylactic ondansetron administration alone was not considered a PONV event.

Covariates included age, sex, body mass index, American Society of Anesthesiologists (ASA) physical status, smoking status, diabetes, hypertension, preoperative hemoglobin, albumin, creatinine, Forced Expiratory Volume in 1 s (FEV1), surgical duration, anesthesia duration, remifentanil infusion rate, total propofol dose, intraoperative blood loss, intraoperative vasoactive drug use, and extent of lung resection. Crystalloid and colloid administration was individualized by the attending anesthesiologist according to clinical assessment; cumulative crystalloid, colloid, and total intraoperative fluid volumes were available for all included patients. Intraoperative vasoactive drug exposure was available only as a binary variable; therefore, drug type, dose, timing, and cumulative exposure could not be modeled.

Because reliable documentation of prior PONV or motion sickness was not available in this retrospective dataset, a complete Apfel score could not be calculated. Sex and smoking status were included as baseline covariates. Postoperative opioid consumption was not included in the primary PONV adjustment set because it occurred after the exposure and could lie on the causal pathway; it was therefore considered when interpreting the PONV findings. Routine ondansetron-based prophylaxis was administered to all patients.

### Statistical analysis

Continuous variables were summarized as mean ± standard deviation or median with interquartile range, as appropriate. Categorical variables were summarized as frequencies and percentages. Patients without an available intraoperative MAP time series were excluded. Within the included cohort, missingness was limited to preoperative hemoglobin (13/420, 3.1%), albumin (12/420, 2.9%), creatinine (20/420, 4.8%), and FEV1 (14/420, 3.3%). Missing values were imputed using the missForest package in R before statistical analysis; no missing values remained in the final analytical dataset. Multivariable linear regression was used for continuous outcomes, and multivariable logistic regression was used for binary outcomes. The primary exposure, MAP <65 mmHg duration, was modeled per 10-min increase. The fully adjusted models included age, sex, body mass index, ASA physical status, smoking status, diabetes, hypertension, preoperative hemoglobin, albumin, creatinine, FEV1, surgical duration, anesthesia duration, remifentanil infusion rate, total propofol dose, intraoperative blood loss, intraoperative vasoactive drug use, and extent of lung resection.

To address the large number of patients with zero hypotension exposure and to assess whether the primary per-10-min model obscured differences between the occurrence and duration of hypotension, two-part sensitivity analyses were performed for 24-h MME and PONV. First, patients with any MAP <65 mmHg exposure were compared with patients without such exposure in the full cohort. Second, among patients with any MAP <65 mmHg exposure, cumulative duration was modeled per 10-min increase. In an additional PONV sensitivity model, 24-h MME was added to the primary adjustment set to explore whether postoperative opioid exposure accounted for part of the observed association. This additional adjustment was exploratory and was not interpreted as a formal mediation analysis. To evaluate potential confounding by intraoperative fluid administration, additional sensitivity models for 24-h MME and PONV added total intraoperative fluid volume to the corresponding adjustment set. A further PONV model included both 24-h MME and total intraoperative fluid volume. These *post hoc* models were interpreted as sensitivity analyses rather than formal mediation analyses.

Sensitivity analyses were performed using hypotension burden below 65 mmHg, time-weighted hypotension below 65 mmHg, minimum MAP, and cumulative MAP <60 mmHg duration. All tests were two-sided, and *P* < 0.05 was considered statistically significant.

### Reporting and ethics

The study was reported in accordance with the Strengthening the Reporting of Observational Studies in Epidemiology (STROBE) statement (29). The study protocol was approved by the Ethics Committee of Shanghai Chest Hospital (approval no. IS26065), and the requirement for informed consent was waived because of the retrospective nature of the study.

## Results

### Study population and baseline characteristics

A total of 420 patients undergoing elective uniportal VATS lung resection were included in the final analytical cohort. Based on cumulative MAP <65 mmHg duration, patients were categorized into no hypotension (*n* = 286), mild-to-moderate hypotension (*n* = 76), and high hypotension burden (*n* = 58) groups. Baseline demographic, preoperative laboratory, surgical, anesthetic, and hemodynamic characteristics are summarized in Table 1.

**Table 1:** Baseline and intraoperative characteristics according to intraoperative hypotension burden.

Characteristic	No hypotension (*n* = 286)	Mild/moderate (*n* = 76)	High burden (*n* = 58)	*P*-value
Age, years	61.6 ± 7.4	63.8 ± 6.9	62.6 ± 6.5	0.056
Male sex, *n* (%)	173 (60.5)	47 (61.8)	30 (51.7)	0.418
Weight, kg	68.3 ± 11.1	68.7 ± 10.6	62.9 ± 9.3	0.002
BMI, kg/m^2^	23.7 ± 3.8	23.9 ± 3.7	21.7 ± 3.3	<0.001
ASA physical status, *n* (%)	II: 175 (61.2); III: 111 (38.8)	II: 40 (52.6); III: 36 (47.4)	II: 31 (53.4); III: 27 (46.6)	0.281
Smoking history, *n* (%)	80 (28.0)	32 (42.1)	20 (34.5)	0.053
Diabetes, *n* (%)	55 (19.2)	17 (22.4)	12 (20.7)	0.823
Hypertension, *n* (%)	158 (55.2)	41 (53.9)	29 (50.0)	0.764
Hemoglobin, g/L	129.1 ± 13.8	131.8 ± 15.6	129.7 ± 13.0	0.339
Albumin, g/L	37.7 ± 4.0	38.7 ± 4.4	37.4 ± 3.8	0.112
Creatinine, μmol/L	77.3 ± 17.2	74.9 ± 15.8	77.1 ± 15.8	0.548
FEV1, L	1.9 ± 0.4	1.7 ± 0.4	1.8 ± 0.4	0.018
Surgical time, min	123.4 ± 26.8	133.5 ± 24.5	127.1 ± 20.9	0.009
Anesthesia time, min	158.5 ± 28.1	166.9 ± 24.5	161.6 ± 22.0	0.049
Remifentanil rate, μg/kg/min	0.171 ± 0.053	0.186 ± 0.060	0.179 ± 0.058	0.080
Propofol total dose, mg	870.5 ± 171.0	889.4 ± 173.7	808.3 ± 166.7	0.017
Blood loss, mL	90.8 ± 51.1	117.5 ± 57.5	125.7 ± 59.4	<0.001
Total intraoperative fluid, mL	1,342.7 ± 542.6	1,598.7 ± 476.2	1,525.9 ± 453.3	<0.001
Resection extent, *n* (%)	Wedge: 67 (23.4); Segmentectomy: 124 (43.4); Lobectomy: 95 (33.2)	Wedge: 8 (10.5); Segmentectomy: 27 (35.5); Lobectomy: 41 (53.9)	Wedge: 4 (6.9); Segmentectomy: 25 (43.1); Lobectomy: 29 (50.0)	<0.001
Intraoperative vasoactive drug, *n* (%)	127 (44.4)	72 (94.7)	56 (96.6)	<0.001
MAP <65 duration, min	0.0 (0.0–0.0)	10.0 (5.0–11.2)	30.0 (21.2–38.8)	<0.001
Minimum MAP, mmHg	74.1 ± 5.0	60.7 ± 3.0	53.7 ± 4.0	<0.001

The mean minimum MAP decreased stepwise across the no-hypotension, mild-to-moderate, and high-burden groups (74.08, 60.71, and 53.71 mmHg, respectively). Intraoperative vasoactive drug use also increased substantially across groups (44.4%, 94.7%, and 96.6%, respectively), suggesting that the hypotension burden categories captured a clinically meaningful gradient of intraoperative hemodynamic instability. Mean total intraoperative fluid volume was 1,342.7 ± 542.6 mL in the no-hypotension group, 1,598.7 ± 476.2 mL in the mild-to-moderate group, and 1,525.9 ± 453.3 mL in the high-burden group (*P* < 0.001). Overall mean crystalloid and colloid volumes were 1,108.3 and 306.0 mL, respectively.

### Distribution of intraoperative hypotension

**Figure 2:**
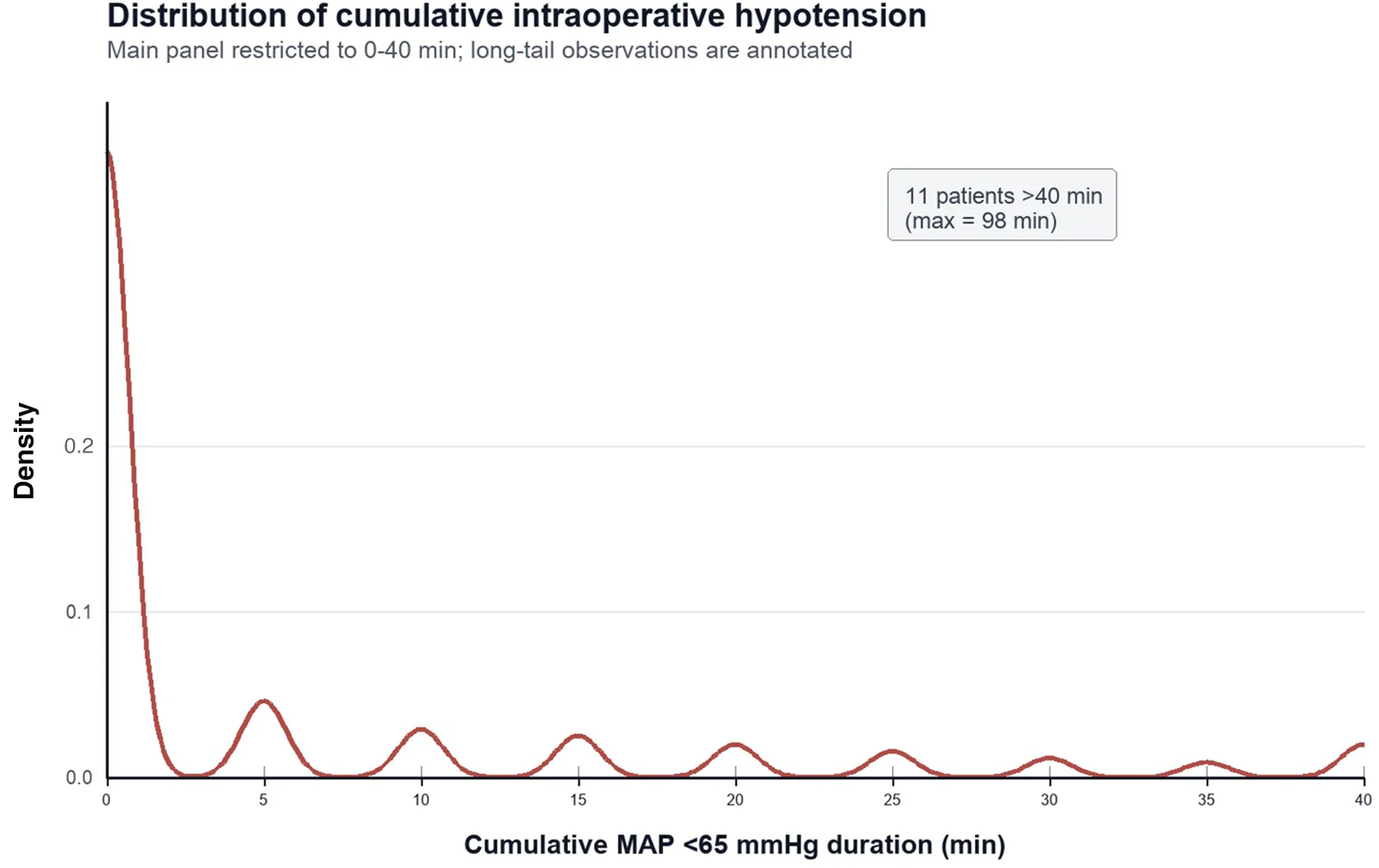
Distribution of cumulative MAP <65 mmHg duration with a kernel density estimate curve and rug plot. The panel is restricted to 0–40 min; 11 patients had durations >40 min (maximum, 98 min). The distribution was zero-inflated and right-skewed.

Overall, 134 patients (31.9%) experienced at least one episode of MAP <65 mmHg during anesthesia. The median cumulative duration of MAP <65 mmHg was 0 min [interquartile range (IQR), 0–5 min], with a mean duration of 6.31 ± 13.25 min and a maximum duration of 98 min. The median hypotension burden below 65 mmHg was 0 mmHg·min (IQR, 0–17.75 mmHg·min), while the mean burden was 37.06 ± 104.75 mmHg·min. The zero-inflated and right-skewed distribution of cumulative hypotension duration is shown in Figure 2.

### Postoperative analgesic consumption and pain profiles across hypotension groups

**Figure 3:**
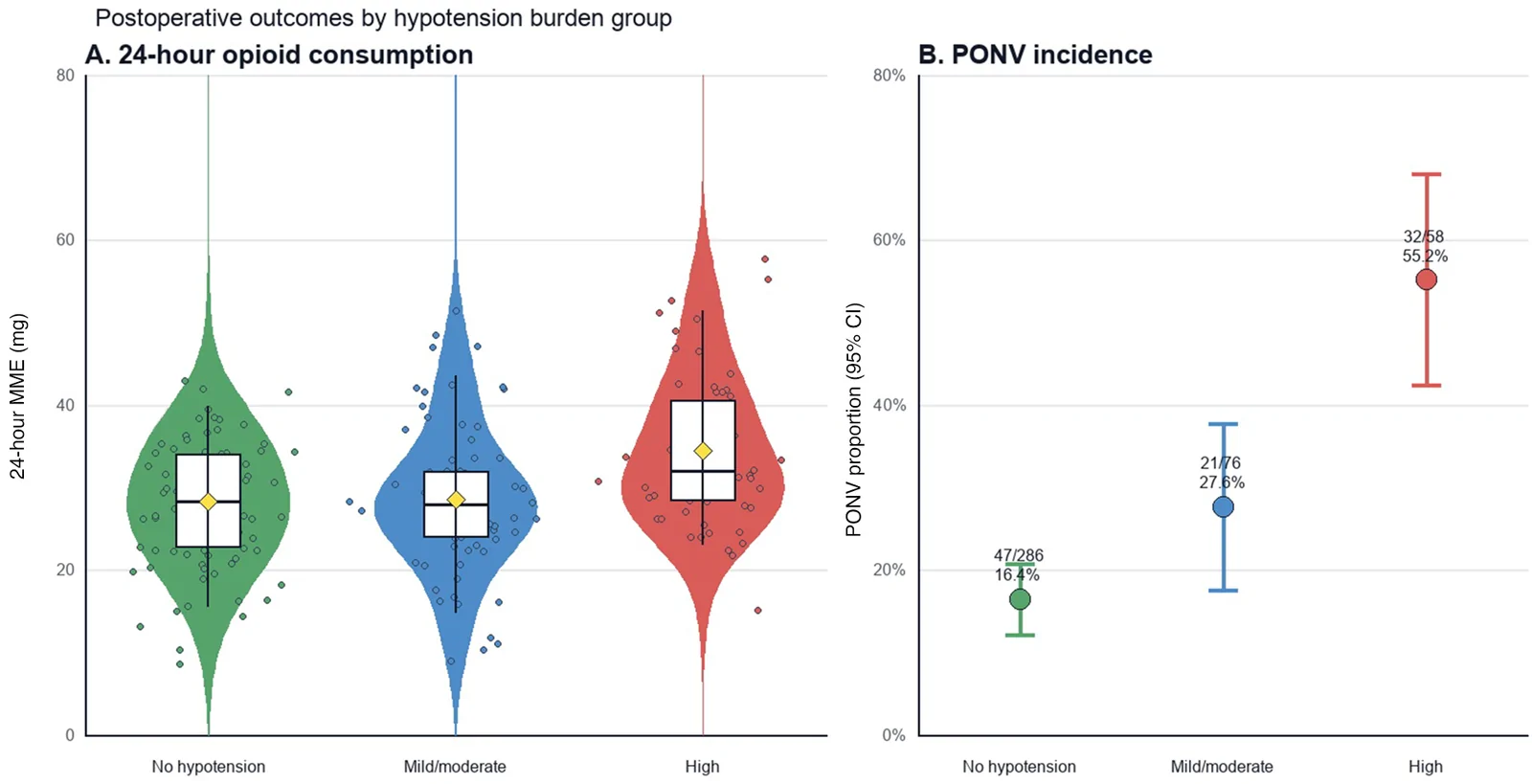
Postoperative outcomes by hypotension burden group. **(A)** Distribution of 24-h intravenous morphine milligram equivalents (MME), shown using violin and box plots. **(B)** Incidence of postoperative nausea and vomiting (PONV) within 24 h, with 95% confidence intervals; labels show event counts/total patients and percentages. Groups comprise no hypotension (*n* = 286), mild-to-moderate hypotension (*n* = 76), and high hypotension burden (*n* = 58).

Postoperative opioid consumption increased progressively with greater hypotension exposure. The mean 24-h MME consumption was 28.24 mg in the no-hypotension group, 28.49 mg in the mild-to-moderate group, and 34.44 mg in the high-burden group (Figure 3). Successful PCA dose deliveries showed a similar gradient across groups (3.60, 3.57, and 4.59, respectively), whereas 24-h resting pain scores were similar across the three groups (2.23, 2.34, and 2.57, respectively).

PONV occurred in 16.4% of patients without hypotension, 27.6% of patients with mild-to-moderate hypotension, and 55.2% of patients with high hypotension burden (Figure 3). Rescue analgesic use showed a less pronounced gradient across groups (21.0%, 26.3%, and 29.3%, respectively).

### Multivariable association between MAP <65 mmHg duration and postoperative outcomes

**Figure 4:**
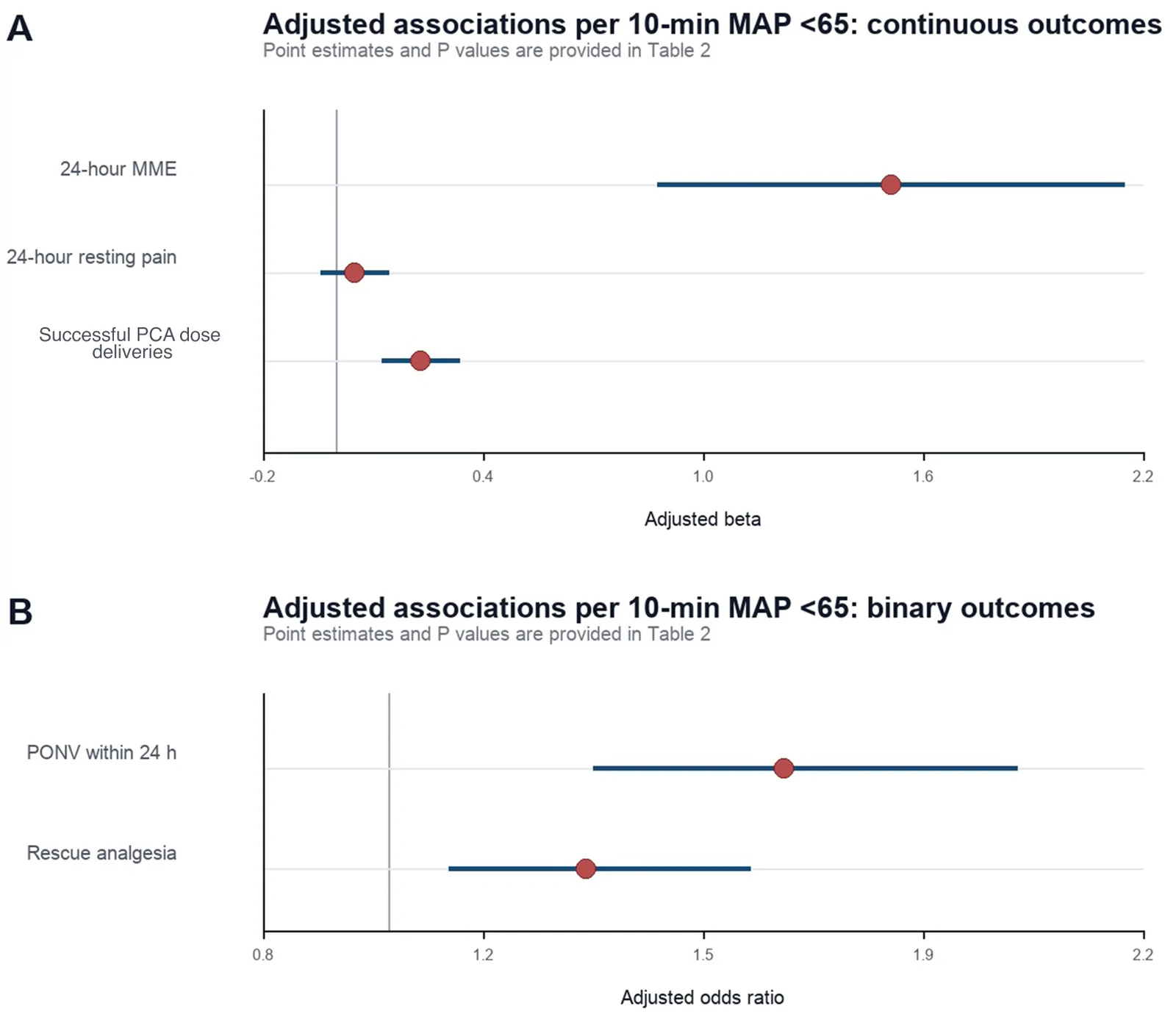
Adjusted associations between cumulative MAP <65 mmHg duration and postoperative outcomes: **(A)** continuous postoperative outcomes; **(B)** binary postoperative outcomes.

After full adjustment, each 10-min increase in MAP <65 mmHg duration was associated with higher 24-h postoperative opioid consumption [*β* = 1.513 mg; 95% confidence interval (CI), 0.877–2.149; *P* < 0.001]. MAP <65 mmHg duration was also associated with more successful PCA dose deliveries within 24 h postoperatively (*β* = 0.230; 95% CI, 0.124–0.336; *P* < 0.001). These adjusted associations for continuous postoperative outcomes are summarized in Table 2 and visualized in Figure 4A.

**Table 2:** Adjusted associations between cumulative MAP <65 mmHg duration and postoperative outcomes.

Outcome	Model	Effect estimate	95% CI	*P*-value
24-h MME	Linear	*β* = 1.513	0.877–2.149	<0.001
24-h resting pain	Linear	*β* = 0.050	−0.043–0.143	0.290
Successful PCA dose deliveries	Linear	*β* = 0.230	0.124–0.336	<0.001
PONV within 24 h	Logistic	OR = 1.628	1.326–1.999	<0.001
Rescue analgesia	Logistic	OR = 1.314	1.096–1.576	0.003

Effect estimates are reported per 10-min increase in cumulative MAP <65 mmHg duration. Fully adjusted models included age, sex, body mass index, ASA physical status, smoking status, diabetes, hypertension, preoperative hemoglobin, albumin, creatinine, FEV1, surgical duration, anesthesia duration, remifentanil infusion rate, total propofol dose, intraoperative blood loss, intraoperative vasoactive drug use, and extent of lung resection. MME, morphine milligram equivalents; MAP, mean arterial pressure; PCA, patient-controlled analgesia; PONV, postoperative nausea and vomiting; CI, confidence interval.

No significant association was observed between MAP <65 mmHg duration and 24-h resting pain score (*β* = 0.050; 95% CI, −0.043–0.143; *P* = 0.290). For binary outcomes, MAP <65 mmHg duration was associated with increased odds of PONV (OR, 1.63; 95% CI, 1.33–2.00; *P* < 0.001) and rescue analgesic use (OR, 1.31; 95% CI, 1.10–1.58; *P* = 0.003). These binary outcome models are summarized in Table 2 and shown in Figure 4B.

### Two-Part, PONV, and fluid-adjusted sensitivity analyses

In the full cohort, the presence of any MAP <65 mmHg exposure was not significantly associated with 24-h MME after adjustment (*β* = 1.785 mg; 95% CI, −0.167–3.737; *P* = 0.073), whereas it was associated with increased odds of PONV (OR, 2.72; 95% CI, 1.52–4.86; *P* < 0.001). Among the 134 patients with any MAP <65 mmHg exposure, each additional 10 min of hypotension was associated with 1.880 mg higher 24-h MME (95% CI, 0.915–2.845; *P* < 0.001) and increased odds of PONV (OR, 1.69; 95% CI, 1.26–2.27; *P* < 0.001). Because only 53 PONV events occurred in this exposed subgroup, the subgroup PONV model was considered exploratory. In the full-cohort PONV model additionally adjusted for 24-h MME, the association with hypotension duration was attenuated but remained significant (OR per 10 min, 1.54; 95% CI, 1.25–1.90; *P* < 0.001). These findings are summarized in Table 3. After additional adjustment for total intraoperative fluid volume, the primary associations remained materially unchanged for 24-h MME (*β* = 1.534 mg per 10 min; 95% CI, 0.893–2.175; *P* < 0.001) and PONV (OR, 1.63; 95% CI, 1.33–2.01; *P* < 0.001). In the model including both 24-h MME and total intraoperative fluid volume, the association with PONV also remained significant (OR, 1.54; 95% CI, 1.25–1.90; *P* < 0.001).

**Table 3:** Two-part, PONV, and fluid-adjusted sensitivity analyses.

Analysis/exposure	Outcome	Adjusted effect	95% CI	*P*-value
Any MAP <65 mmHg vs. none (*N* = 420)	24-h MME	*β* = 1.785 mg	−0.167–3.737	0.073
Any MAP <65 mmHg vs. none (*N* = 420)	PONV within 24 h	OR=2.723	1.525–4.862	<0.001
MAP <65 mmHg duration per 10 min among exposed patients (*n* = 134)	24-h MME	*β* = 1.880 mg	0.915–2.845	<0.001
MAP <65 mmHg duration per 10 min among exposed patients (*n* = 134)	PONV within 24 h	OR = 1.689	1.258–2.268	<0.001
MAP <65 mmHg duration per 10 min, additionally adjusted for 24-h MME (*N* = 420)	PONV within 24 h	OR = 1.541	1.249–1.901	<0.001
MAP <65 mmHg duration per 10 min + total fluid volume (*N* = 420)	24-h MME	*β* = 1.534 mg	0.893–2.175	<0.001
MAP <65 mmHg duration per 10 min + total fluid volume (*N* = 420)	PONV within 24 h	OR = 1.632	1.328–2.006	<0.001
MAP <65 mmHg duration per 10 min + 24-h MME + total fluid volume (*N* = 420)	PONV within 24 h	OR = 1.542	1.249–1.904	<0.001

All models were adjusted for the same covariates as the primary analyses. Fluid-adjusted models additionally included total intraoperative fluid volume. The exposed-only PONV model was exploratory because 53 events were available for 20 model parameters. Models additionally adjusted for 24-h MME were not interpreted as formal mediation analyses. MME, morphine milligram equivalents; MAP, mean arterial pressure; PONV, postoperative nausea and vomiting; CI, confidence interval.

### Sensitivity analyses

**Figure 5:**
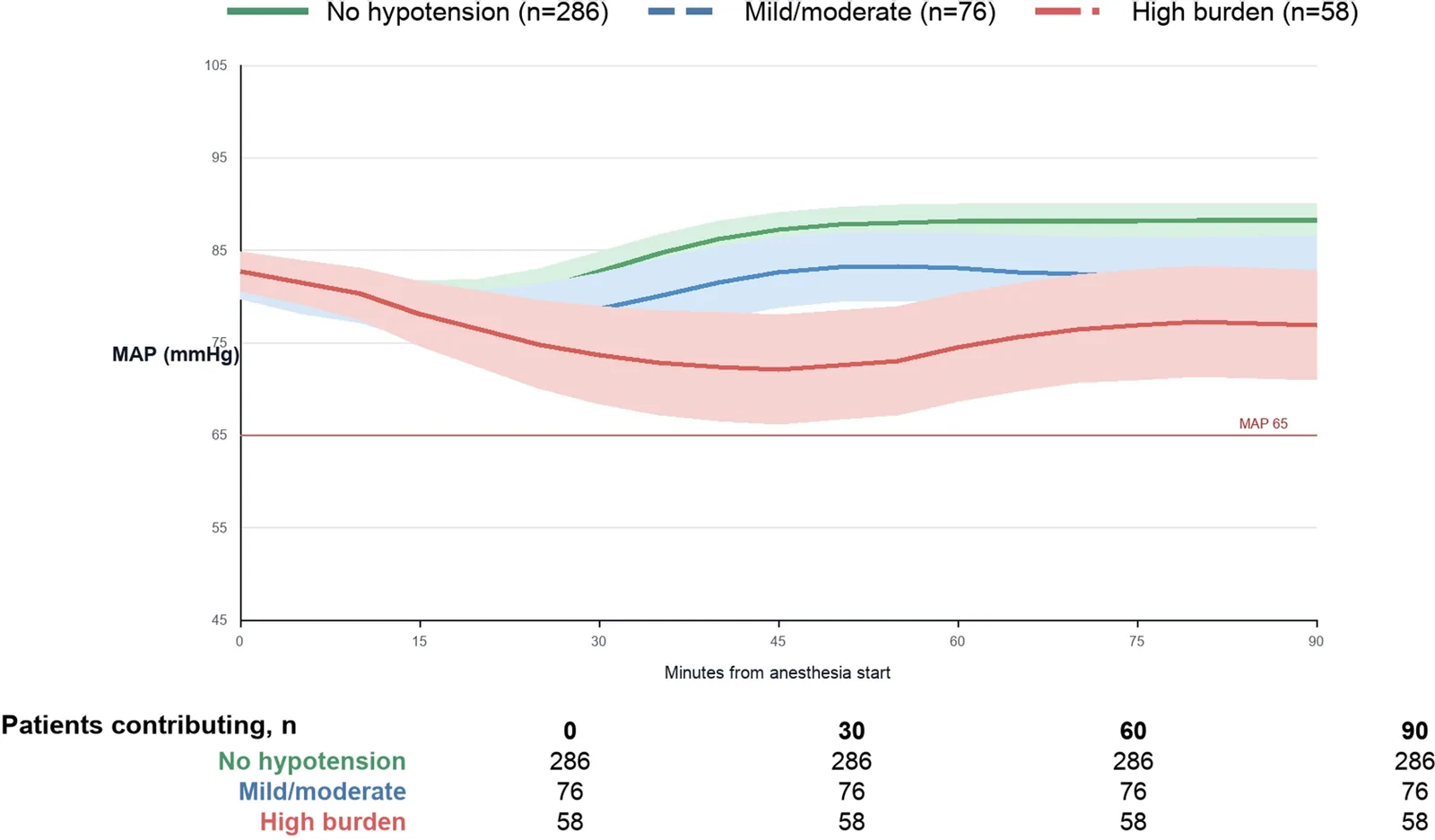
Mean intraoperative MAP trajectories during the first 90 min after anesthesia start by hypotension burden group. Lines show group means and bands show pointwise 95% confidence intervals. Numbers contributing at 0, 30, 60, and 90 min are shown below; all patients contributed throughout (*n* = 286, 76, and 58).

Sensitivity analyses using alternative hypotension metrics yielded broadly consistent findings for PCA behavior and PONV. Hypotension burden below 65 mmHg was associated with more successful PCA dose deliveries and increased odds of PONV. Time-weighted hypotension below 65 mmHg and cumulative MAP <60 mmHg duration showed directionally consistent associations with successful PCA dose deliveries and PONV. The mean intraoperative MAP trajectories during the first 90 min, with pointwise 95% confidence intervals and time-specific sample sizes, are presented in Figure 5.

## Discussion

In this single-center cohort of patients undergoing elective uniportal VATS lung resection, greater cumulative duration of intraoperative MAP <65 mmHg was associated with higher 24-h opioid consumption, more successful PCA dose deliveries, increased odds of PONV, and higher likelihood of rescue analgesic use after multivariable adjustment. In contrast, the 24-h resting pain score was not significantly associated with hypotension duration. Sensitivity analyses using alternative hypotension metrics, including hypotension burden below 65 mmHg, time-weighted hypotension, and cumulative MAP <60 mmHg duration, showed broadly consistent directions of effect for PCA behavior and PONV. These findings extend prior perioperative blood pressure literature, which has mainly emphasized organ injury and mortality outcomes, toward early recovery endpoints after thoracic surgery (15–20). The two-part sensitivity analyses further indicated that the occurrence of any hypotension alone was not significantly associated with 24-h MME, whereas longer hypotension duration among exposed patients remained associated with higher opioid consumption. The association with PONV was modestly attenuated but persisted after additional adjustment for 24-h MME. The associations with 24-h MME and PONV were stable after additional adjustment for total intraoperative fluid volume.

The observed association between MAP <65 mmHg duration and 24-h opioid consumption was statistically significant but modest in absolute magnitude: approximately 1.5 mg of additional intravenous morphine equivalent per 10 min of hypotension in the primary model. Whether a difference of this magnitude is clinically perceptible in routine thoracic practice is uncertain, particularly because no corresponding association was observed with resting pain at 24 h. The estimate should therefore be interpreted as a population-level signal of greater analgesic requirement rather than evidence that hypotension alone is a dominant determinant of postoperative pain. Thoracic surgical pain is multifactorial, and regional analgesia, incision and chest drain factors, extent of resection, and patient vulnerability remain central drivers (4–8). Successful PCA dose deliveries provide complementary information about analgesic demand, but their clinical importance should likewise be interpreted in the context of the individualized PCA regimen and the absence of dynamic pain measurements (1, 8, 9).

One possible explanation is that hypotension burden functions as an integrated marker of intraoperative physiologic stress. MAP values below 65 mmHg have been repeatedly associated with organ injury after non-cardiac surgery, and both threshold-based and burden-based approaches are supported by perioperative blood pressure literature (15–20). Although postoperative pain is distinct from renal or myocardial injury, the duration and depth of impaired perfusion could plausibly interact with surgical manipulation, one-lung ventilation, inflammatory activation, and nociceptive processing (21–23). However, no inflammatory biomarkers, perfusion markers, or measures of tissue injury were available in this study. These mechanisms therefore remain speculative and hypothesis-generating and are not supported directly by the present data.

The dissociation between successful PCA dose deliveries and 24-h resting pain score deserves attention. Resting pain at 24 h may be blunted by ongoing PCA use, rescue analgesia, and recovery from the most intense immediate postoperative stimulus, whereas successful PCA dose deliveries may better capture patient demand for analgesia. This pattern is consistent with procedure-specific VATS analgesia guidance, which emphasizes early multimodal and regional analgesia while recognizing that pain after VATS can remain clinically important despite minimally invasive access (8, 9). Because all patients in the present pathway received thoracic paravertebral block, the findings also suggest that hemodynamic variables may contribute to residual variability even under a standardized regional analgesic strategy (10–12). Remifentanil exposure and postoperative opioid use may further complicate this relationship because acute opioid tolerance and opioid-induced hyperalgesia can increase postoperative pain and morphine requirement (24–27).

The association between hypotension duration and PONV is also plausible but should be interpreted cautiously. Female sex, non-smoking status, prior PONV or motion sickness, volatile anesthetic exposure, and postoperative opioid use are established predictors of PONV, and validated risk models such as the Apfel score remain the standard framework for clinical risk assessment (30). The current dataset did not include reliable documentation of prior PONV or motion sickness, and all patients received ondansetron-based prophylaxis. The observed relationship may therefore reflect several pathways, including an indirect pathway through greater postoperative opioid exposure, unmeasured baseline susceptibility, or common intraoperative causes of both hypotension and PONV. The present observational analysis cannot distinguish a direct association from an opioid-mediated pathway. The finding should therefore be interpreted as hypothesis-generating rather than evidence that hypotension itself causes PONV. PONV remains clinically relevant because it can delay oral intake, reduce satisfaction, and interfere with enhanced recovery after lung surgery (1, 31, 32).

These results should not be read as proof that raising intraoperative blood pressure will reduce postoperative pain or PONV. Observational associations are vulnerable to residual confounding and confounding by indication. Patients with greater hypotension burden also had higher vasoactive drug use and, in some comparisons, longer procedures, greater blood loss, more extensive resections, or greater fluid administration. Total intraoperative fluid volume was available and was included in sensitivity analyses, but the timing of fluid administration, fluid responsiveness, and the relationship between individual boluses and hypotension episodes could not be evaluated. Vasoactive exposure was available only as a binary variable, and drug type, dose, timing, and duration could not be modeled. Anesthetic depth, nociception monitoring, and the exact timing of hypotension relative to incision, one-lung ventilation, or emergence were also unavailable. These limitations prevent causal inference and should guide the design of future prospective studies.

Additional limitations concern cohort selection, outcome ascertainment, and exposure modeling. Of the 612 patients assessed for eligibility, 48 were excluded because core records or 24-h outcomes were missing, and 19 because the intraoperative MAP time series was incomplete. Because included and data-related excluded patients were not compared, selection bias cannot be excluded. Exposure reconstruction used routinely charted values at approximately 5-min intervals rather than continuous waveform data; brief events may therefore have been missed, and classification depended on charting fidelity. Pain assessment was limited to resting pain at a single 24-h time point; dynamic pain during coughing or movement was not collected because of the retrospective study design. This limits characterization of functionally relevant pain after thoracic surgery. In addition, most patients had no recorded MAP <65 mmHg exposure and positive durations were right-skewed. The per-10-min coefficient should therefore be interpreted as an average model-based association that may not capture a threshold or other non-linear relationship. The PONV model restricted to the 134 patients with hypotension included only 53 events relative to the number of adjusted covariates and should therefore be regarded as exploratory and potentially vulnerable to overfitting.

Future work should prospectively evaluate intraoperative blood pressure trajectories alongside anesthetic depth, nociception indices, vasopressor and fluid therapy, regional block performance, inflammatory markers, and dynamic postoperative pain endpoints. A time-resolved approach may help determine whether hypotension during specific surgical phases is more strongly associated with early analgesic demand than cumulative duration alone. Interventional trials will be required to test whether individualized arterial pressure management can improve analgesic recovery, reduce opioid exposure, or lower PONV after VATS. Until such evidence is available, prolonged hypotension should be regarded as a potential marker of less favorable early recovery rather than a proven treatment target. Optimal VATS recovery continues to depend on multimodal analgesia, regional anesthesia, opioid stewardship, and structured PONV prophylaxis.

## Data Availability

The datasets presented in this article are not readily available, but the datasets generated during and/or analyzed during the current study are available from the corresponding author on reasonable request. Requests to access the datasets should be directed to wu_jingxiang@sjtu.edu.cn.
